# A comprehensive analysis of erectile dysfunction prevalence and the impact of prostate conditions on ED among US adults: evidence from NHANES 2001-2004

**DOI:** 10.3389/fendo.2024.1412369

**Published:** 2025-01-13

**Authors:** Yuhao Zhang, Nan Zang, Yingyue Xiang, Fanlu Lin, Xue Liu, Jing Zhang

**Affiliations:** ^1^ Department of Endocrinology, Qilu Hospital of Shandong University, Jinan, Shandong, China; ^2^ Department of Urology, Linyi Central Hospital, Linyi, Shandong, China; ^3^ The Second Department of Infectious Disease, Shanghai Fifth People’s Hospital, Fudan University, Shanghai, China; ^4^ Center of Community-Based Health Research, Fudan University, Shanghai, China

**Keywords:** benign prostatic hyperplasia (BPH), prostatitis, prostatic cancer (Pca), erectile dysfunction (ED), National Health and Nutrition Examination Survey (NHANES)

## Abstract

**Background:**

Erectile dysfunction (ED) is characterized by the inability to achieve or maintain penile erection sufficient for intercourse. While previous research suggests a potential link between ED and prostate pathologies, the association between benign prostatic hyperplasia (BPH), prostatitis, prostatic cancer (PCa), and ED remains to be elucidated.

**Methods:**

Data from participants (40-80 years, n=2225) were extracted from the NHANES 2001-2004 for this observational study. The investigation encompassed the following aspects: assessment of ED prevalence within subgroups, comparison of baseline characteristics between individuals with and without ED, analysis of associations between BPH, prostatitis, PCa, and ED using multivariable weighted logistic regression in the 40-60 and 60-80 age groups and subgroup analysis based on body mass index, hypertension, diabetes, and smoking status.

**Results:**

Among the 2225 participants, the weighted prevalence of ED was 27.47%, with 16.17% in the 40-60 years age group and 56.98% in the 60-80 years age group. BPH had an ED prevalence of 47.57%, prostatitis 34.62%, and PCa 85.62%. Comparative analysis between ED and non-ED groups revealed significant differences in education levels, PIR, smoking and alcohol status, creatinine, total cholesterol, LDL cholesterol, diabetes, hypertension, BPH, and PCa. Multivariate logistic regression analysis identified BPH as an independent risk factor for ED in the 60-80 years age group (OR=1.93; 95% CI, 1.18-3.18, P=0.02), and PCa was positively associated with ED in both the 40-60 years group (OR=11.90; 95% CI, 1.41-100.50, P=0.03) and the 40-80 years group (OR=7.30; 95% CI, 2.12-25.08, P=0.01). No clear correlation was found between prostatitis and ED. Subgroup analyses indicated that the association between BPH and ED was significant in non-diabetic, overweight/obese, and smoking groups, while the association between PCa and ED was more pronounced in non-diabetic, hypertensive individuals across all body mass index (BMI) categories, and in both smoking and non-smoking groups. Prostatitis showed no significant relationship with ED in any subgroup.

**Conclusion:**

The study established BPH and PCa as significant risk factors for ED, with no substantial link detected between prostatitis and ED. This finding highlights the necessity for tailored screening and management protocols for individuals with BPH and PCa to mitigate the burden of ED.

## 1 Introduction

Erectile Dysfunction (ED), also known as insufficient penis erection which was defined as the inability to obtain or maintain a sufficient erect penis to complete sexual activity, is a traditional male dysfunction (1–3). The prevalence of ED varies across continents. In North America, particularly the United States, the prevalence of ED is well-documented. The Massachusetts Male Aging Study (MMAS) reported that approximately 52% of men aged 40-70 experience some degree of ED (4). In Europe, the prevalence of ED is similar to that in North America. The European Male Aging Study (EMAS) found that about 19% of men aged 40-79 experience moderate to severe ED, with prevalence increasing with age (5). In Asia, the prevalence of ED varies widely. In China, studies indicate that approximately 26% of men aged 40-70 experience ED (6). However, the prevalence is slightly lower in Japan, with about 10-20% of men affected. Data on ED in Africa is less comprehensive, but available studies suggest a prevalence of around 15-30% among men aged 40 and above. Factors such as limited healthcare access and chronic diseases like diabetes and hypertension contribute to these figures (7). In Australia, the prevalence of ED is similar to that in other Western countries, with studies indicating that about 40% of men over 40 experience some form of ED (8). The prevalence increases with age, affecting up to 60% of men over 70. Globally, it is generally believed that the incidence of ED increases with the age of men (9, 10). The landmark Study called MMAS, conducted in 1994, provided a valuable insight: the prevalence of mild to moderate ED was 52% in men aged 40-70 years, while the incidence of severe (complete) ED increased rapidly from 5% to 15% with men aged (4). In fact, the true incidence of ED may be much higher than this study concluded, because some patients would not want to seek medical help for unspeakable reasons.

Clinical research on ED has seen rapid advancement in recent years. Previous epidemiological studies have shown a significant correlation between the presence of prostate-related health issues and increased incidence of ED (11). Similarly, prostate diseases such as BPH and prostate cancer are among the most common medical conditions in aging men. Meanwhile, the diagnosis and subsequent treatment of prostate diseases can lead to psychological distress, anxiety, and depression, conditions known to exacerbate or even precipitate ED (12). These suggest a multifactorial relationship that warrants further exploration to improve clinical management strategies. Since the prevalence of ED is rapidly increasing, it is particularly important to find out the risk factors related to the occurrence of ED. In the past, ED was considered a psychological disorder, but recent studies have shown that ED is a multi-dimensional and relatively common male dysfunction (10). BPH has become a more common disease in the elderly male population, and its diseases incidence rate currently up to more than 50% of men over 50 years of age (13). Previous studies have suggested that Benign prostatic hyperplasia (BPH) and ED may be related (14, 15). Park HJ et . proved that approximately 70% of men with BPH had ED, with severity of one disease often correlating with the other (16). Additionally, much evidence has suggested that BPH is related with ED, which could be interpreted in the field of intestinal microbiota (17). Study focusing on the mechanism of vascular damage have indicated that BPH and ED have common etiological factors (18).

In prostate cancer patients, many of them face difficulties in sexual life, including ED. Published study indicated that over 70% of men perceived negative impacts on their sexual health following PCa diagnosis and treatment (19). Over the past decade, there has been considerable interest in the potential association between PCa and ED, with the increase in published articles and media coverage on this.

Based on the anatomical and physiological interplay between the prostate and structures essential for erectile function, there is a significant association between prostate diseases (such as BPH, prostatitis, and prostate cancer) and the prevalence of ED (11). And the treatment modalities for prostate diseases, including surgical and pharmacological interventions, significantly influence the occurrence and severity of ED. Grounded in existing literature, we suggest that there is a physiological and clinical link between prostate disease and erectile function.

While there were several researches on ED and prostate diseases individually, there is a lack of comprehensive studies examining their interrelation. By quantifying the association between prostate diseases and ED, our study aims to elucidate the multifaceted mechanisms underlying these co-occurring conditions. The ultimate goal is to inform and enhance therapeutic approaches, thereby improving quality of life for affected individuals. In this study, we used the data of the population participating in the National Health and Nutrition Examination (NHANES) study to determine the correlation between BPH, prostatitis, PCa and ED.

## 2 Materials and methods

### 2.1 Study population in NHANES

**Figure 1:**
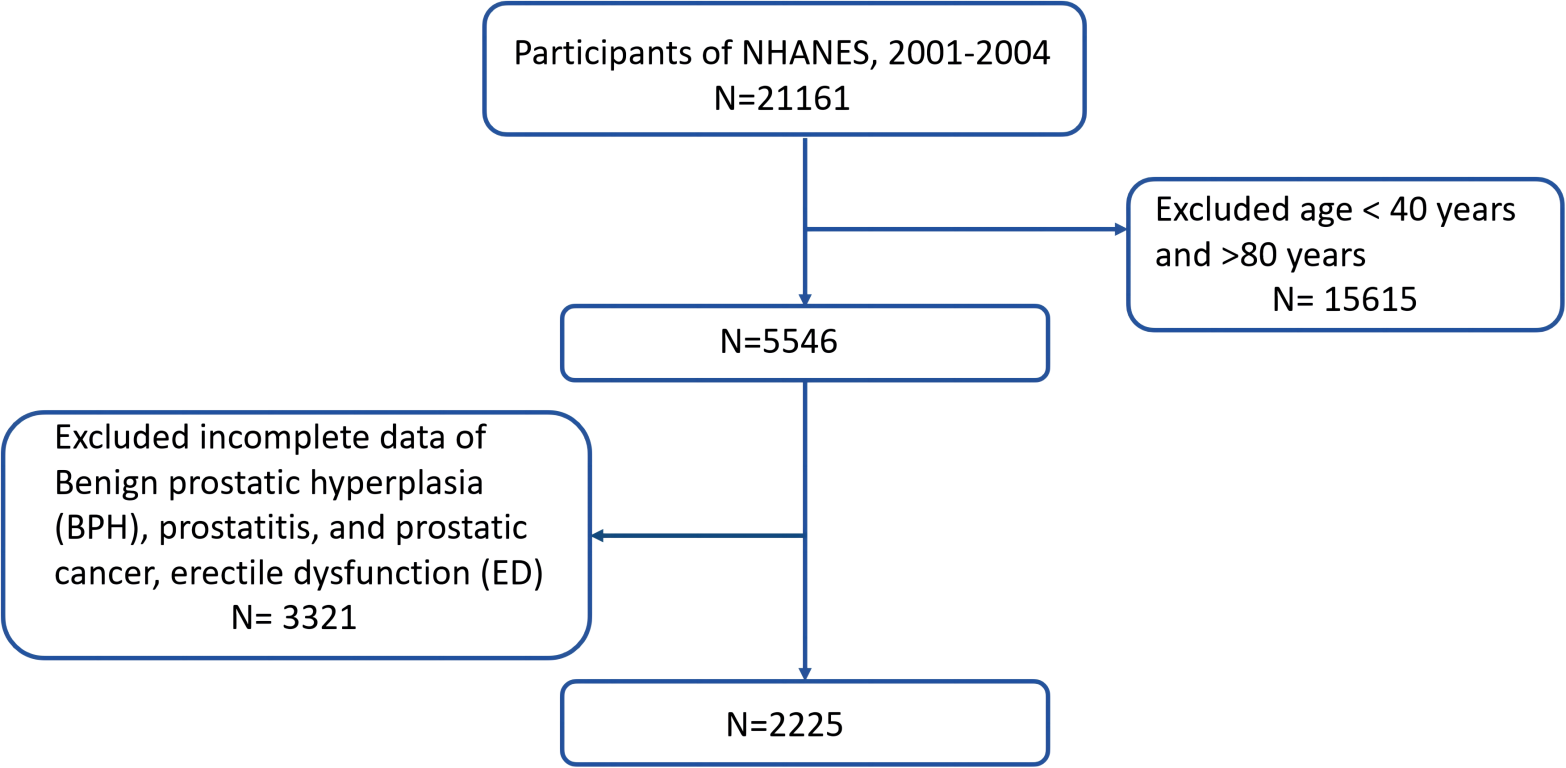
Study flowchart National Health and Nutrition Examination Survey (NHANES), 2001- 2004.

The data which used in the current research is publicly available through the NHANES database (https://www.cdc.gov/nchs/nhanes/index.htm) (20). The NHANES study protocols were approved by the Research Ethics Review Board of NCHS, and informed consent was obtained from all participants (21). We merged two cycles of NHANES data from 2001 to 2004 for this research (N = 21161). The exclusion criteria were as follows: (i) men aged 80 years, (ii) men who did not know the answer, refused to answer the question, or had a missing value to the question “Enlargement was BPH”, “Have an infection or inflammation of the prostate gland at the present time? “, “Have you ever been told by a doctor or health professional that you had prostate cancer? “ and “How would you describe your ability to obtain and maintain an erection sufficient for satisfactory sexual intercourse”. Finally, we enrolled 2225 participants. **Figure 1** illustrates the complete procedure of integrating the data.

### 2.2 Definition and assessment of BPH and ED in NHANES

Men who responded “yes” to the question “Enlargement was BPH” were categorized as having been diagnosed with BPH, who responded “yes” to the question “ Have an infection or inflammation of the prostate gland at the present time?” were categorized as having been diagnosed with prostatitis, who responded “yes” to the question “ Have you ever been told by a doctor or health professional that you had prostate cancer?” were categorized as having been diagnosed with PCa.

According to the Massachusetts Male Aging Study(MMAS) (22), the adult men in the study were asked the next issues through a questionnaire on erectile function: “How would you describe your ability to obtain and maintain an erection sufficient for satisfactory sexual intercourse?”. The answers included “always or almost always able,” “usually able,” “sometimes able,” and “never able”. Based on the previous fundings (23, 24), having ED was defined as men who responded “sometimes able” or “never able” to maintain erectile function, while participants who responded “nearly always able” or “usually able” were defined as not having ED.

### 2.3 Acquisition of covariates used in NHANES

Data on individuals’ social-demographic factors, health-related status and health behaviors were gathered through interviews based on questionnaires and Mobile Examination Centers (MECs).

Social-demographic factors included age (years), race (Mexican American, Non-hispanic black, Non-hispanic white, Other hispanic, Other race), education levels (under high school, high School or Equivalent, above high school), marry status (never married, married, divorced/widowed) and poverty income ratio (PIR). Based on Supplemental Nutrition Assistance Program eligibility, PIR was classified as the ratio of family income to poverty into <1.30, 1.30−3.49, and ≥3.50.

Health-related status was represented by body mass index (BMI) (kg/m^2^). The BMI was computed as the weight divided by the square of the height.

One of the health behaviors was smoking (never smoker, former smoker, and now smoker). Never smokers were defined as individuals who smoked less than 10 cigarettes in their entire lives. Those who had smoked 100 cigarettes or more in a period of time during their lives were defined as former smokers if their response was “No” when they were asked the question “Do you smoke now?”, or else they were defined as current smokers if their response was “Yes” (25). The alcohol status in our study was divided into three distinct groups. “Never” drinkers were classified as individuals who had consumed less than 12 drinks in any one year. “Former” drinkers were categorized as those who had consumed at least 12 drinks in any one year but currently not drinking. Lastly, “current” drinkers were classified as individuals who had consumed at least 12 drinks in any one year and currently drinking (26). In terms of current drinking status, we established specific definitions for current heavy alcohol users and current moderate alcohol users. Current heavy alcohol users were identified as individuals who consumed at least 3 drinks per day for females, 4 drinks per day for males, or engaged in binge drinking on 5 or more days per month. On the other hand, current moderate alcohol use was defined as consuming at least 2 drinks per day for females, 3 drinks per day for males, or engaging in binge drinking on at least 2 days per month (26).

Venous blood samples were taken to measure creatinine (Cr, mg/dl), uric acid (mol/L), triglyceride (mmol/L), LDL cholesterol (mmol/L), HDL cholesterol (mmol/L) and total cholesterol (mmol/L).

One of the medical history included Diabetes Mellitus (DM) and hypertension. DM was defined as a glycohemoglobin level of ≥ 6.5%, the use of diabetes medication or insulin, or a self-reported diagnosis of diabetes (27). Hypertension was defined as the use of antihypertensive medications, a medical diagnosis of hypertension, or three consecutive measurements of systolic blood pressure at ≥140 mmHg or diastolic blood pressure at≥90 mmHg (28).

### 2.4 Statistical analysis

The determination of weights for analysis followed the guidelines outlined in the NHANES database. Baseline characteristics were presented using the weighted mean and standard error (SE) for continuous variables and weighted proportions for categorical variables. Weighted multivariate logistic regression models were employed to assess the odds ratio (OR) and 95% confidence interval associated with IR. In model 1, no adjustments were made for any variable. In contrast, model 2 involved adjustments for social-demographic factors and health behaviors (education level, race, marry status, PIR, smoke status and alcohol status). Model 3 further incorporated adjustments for creatinine, uric acid, triglyceride, total cholesterol, HDL cholesterol, LDL cholesterol, DM and hypertension.

For cases with missing covariates, we created imputed datasets using chained equations. The “mice” R package was utilized for multiple imputations on samples with incomplete covariate information. A significance threshold of P < 0.05 was used to determine statistical significance. All analyses of the National Health and Nutrition Examination Survey (NHANES) data took into account the complex survey design, using weighted analysis with the survey package in R software (version 4.3.2).

## 3 Results

### 3.1 The prevalence of ED

Among 2225 participants, the weighted ED prevalence is 27.47% (95% CI, 27.45%-27.49% [n = 801]). Specifically, the prevalence of ED in the 40-60 age group is 16.17% (95% CI, 14.91%-17.43%); while in the 60-80 age group, the prevalence of ED is 56.98% (95% CI, 55.38%-58.58).

Overall, the prevalence of ED is more significant among individuals of other Hispanic ethnicity, married individuals, those with a PIR<1.3, individuals with under high school education levels, former-smokers, former-drinkers, individuals were under weight, as well as those with diabetes and hypertension. Slightly differently, in the 40-60 age group, the prevalence of ED is higher among never married individuals, now smokers and obese. While in the 60-80 age group, the prevalence of ED is higher among individuals of Non-Hispanic Black and divorced/widowed individuals (see **Table 1**).

**Table 1:** Comparison of ED prevalence between the two groups of patients with 40-60 and 60-80 age.

	Total	40-60	60-80
	27.47(0.02)	16.17(1.26)	56.98(1.60)
Race			
**Mexican American**	23.50(3.82)	15.39(3.40)	59.10(4.50)
**Non-Hispanic Black**	28.68(2.26)	18.88(2.63)	61.80(3.19)
**Non-Hispanic White**	27.40(1.27)	15.14(1.31)	56.93(1.82)
**Other Hispanic**	36.28(8.22)	31.93(7.93)	58.92(15.56)
**Other Race**	20.14(5.06)	10.84(6.20)	43.46(8.59)
Marital status			
**Never married**	22.52(3.41)	19.82(3.75)	42.59(10.62)
**Married**	27.82(1.31)	16.42(1.43)	56.05(1.99)
**Divorced/Widowed**	27.79(2.67)	13.54(2.65)	64.42(3.35)
Poverty Income Ratio			
**<1.30**	35.92(3.52)	24.68(3.75)	64.89(4.22)
**1.30-3.49**	21.74(1.51)	17.84(1.73)	61.55(2.51)
**>=3.50**	33.91(1.89)	13.47(1.55)	50.41(2.60)
Education levels			
**Under High School**	54.42(4.73)	37.17(5.80)	69.70(4.50)
**High School or Equivalent**	29.10(1.64)	16.81(1.91)	57.91(2.64)
**Above High School**	23.47(1.35)	14.38(1.65)	53.06(2.01)
Smoke status			
**Never**	21.77(1.74)	13.40(1.54)	51.12(3.08)
**Former**	34.42(2.07)	16.48(2.71)	60.76(2.49)
**Now**	25.39(2.56)	19.74(2.98)	54.68(4.30)
Alcohol status			
**Never**	29.83(3.66)	12.89(3.44)	56.34(6.11)
**Former**	37.65(2.88)	24.45(3.46)	60.41(3.56)
**Mild**	27.06(1.84)	14.34(1.81)	59.26(2.80)
**Moderate**	19.15(2.84)	10.77(2.78)	48.01(6.61)
**Heavy**	18.61(2.92)	15.23(3.31)	40.80(5.72)
BMI			
**Under weight**	34.22(15.63)	17.74(15.77)	89.52(10.80)
**Normal weight**	26.09(3.14)	15.50(3.13)	54.39(4.14)
**Over weight**	24.37(1.20)	12.95(1.58)	52.71(3.81)
**Obese**	32.56(2.59)	20.88(2.57)	64.75(2.75)
DM			
**Yes**	53.71(3.46)	38.95(5.21)	72.99(3.76)
**No**	46.29(3.46)	13.29(1.11)	52.33(1.88)
Hypertension			
**Yes**	37.52(1.84)	22.13(2.49)	61.24(1.87)
**No**	19.28(1.27)	12.58(1.46)	49.47(2.97)
Prostate Conditions			
**Benign prostatic hyperplasia (BPH)**	47.57(2.97)	30(20.63)	188(65.95)
**Prostatitis**	34.62(9.38)	4(19.32)	10(77.90)
**Prostatic cancer (PCa)**	85.62(4.92)	4(79.16)	51(86.67)

The bold values were used to emphasize the statistical analysis results of P < 0.05, that is, the results were statistically significant.

### 3.2 Baseline characteristics of participants according to ED

**Figure 2:**
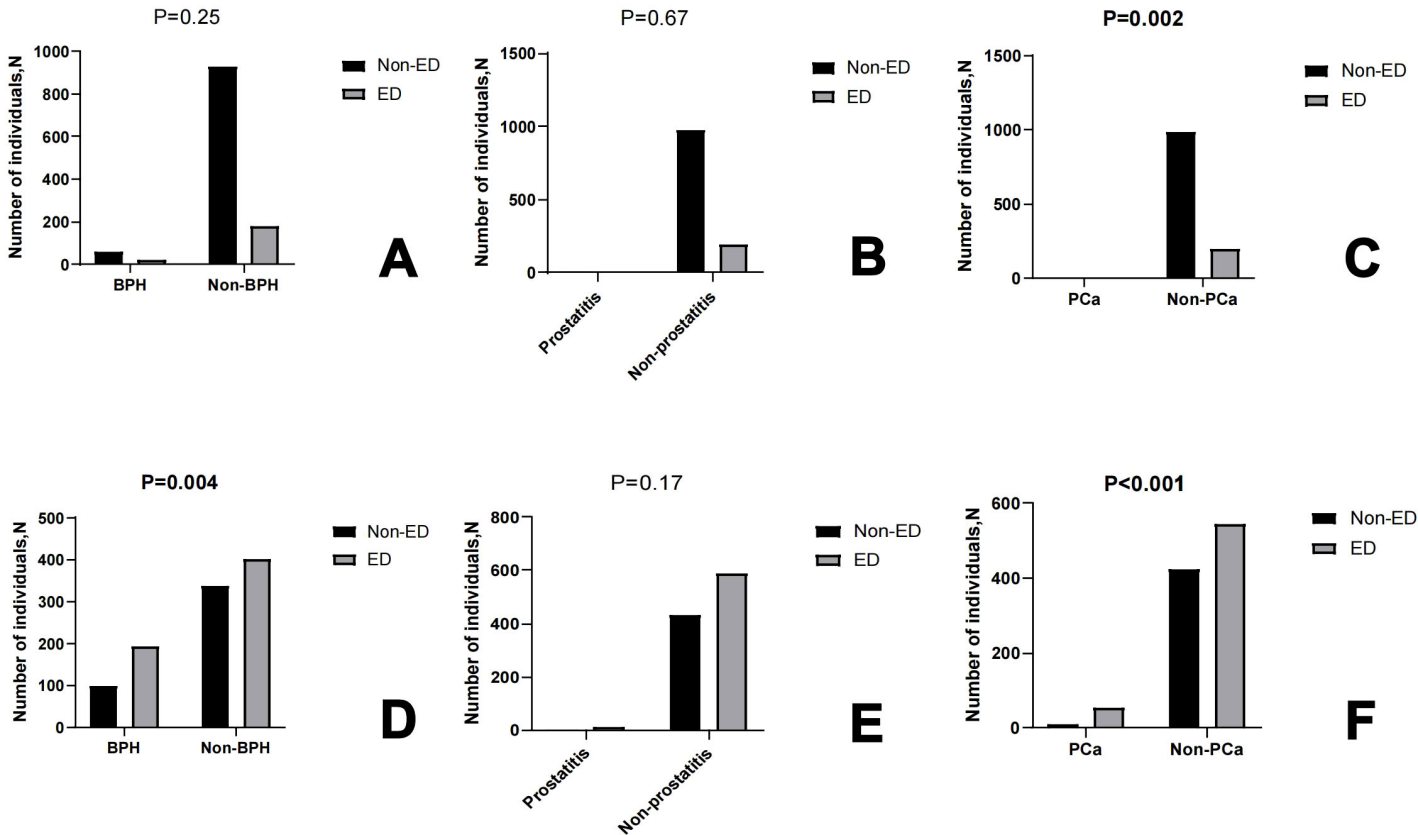
The box graph shows the number of individuals Non-ED and ED based on BPH or not, prostatitis or not, PCa or not in 40-60 age **(A–C)** and 60-80 age **(D–F)**, respectively.

All participants are categorized into two groups based on ED or not: ED group (N = 801) and Non-ED group (N= 1424). There is a significant difference in education levels, PIR, smoke status, alcohol status, creatinine, total cholesterol, LDL cholesterol, diabetes or not, hypertension or not, BPH or not and PCa or not (P<0.05) (see **Table 2**). Furthermore, we analyzed the baseline characteristics of the 40-60 and 60-80 age groups separately (see **Supplementary Table 1**). At the same time, we conducted an analysis on the prevalence of ED based on the presence or absence of BPH, prostatitis, and PCa in the 40-60 age group and the 60-80 age group respectively. The results showed that in the 40-60 age group, there is a statistically significant difference in the prevalence of ED between patients with and without PCa (P=0.002). In the 60-80 age group, differences in the prevalence of ED exist between patients with and without BPH, as well as between patients with and without PCa. There is no significant statistical difference in the prevalence of ED between patients with and without prostatitis in both age groups. (see **Figure 2**).

**Table 2:** The basic characteristics based on ED or not.

	Total (N = 2225)	Non-ED (N = 1424)	ED (N = 801)	P value
**Race**				0.30
**Mexican American**	423(19.01)	273(4.96)	150(4.03)	
**Non-Hispanic Black**	423(19.01)	273(8.71)	150(9.24)	
**Non-Hispanic White**	1255(56.4)	799(79.40)	456(79.12)	
**Other Hispanic**	70(3.15)	40(3.58)	30(5.38)	
**Other Race**	54(2.43)	39(3.35)	15(2.23)	
**Education levels**				**< 0.0001**
**Under High School**	347(15.6)	166(4.15)	181(13.09)	
**High School or Equivalent**	810(36.4)	512(33.91)	298(36.75)	
**Above High School**	1068(48)	746(61.94)	322(50.17)	
**Marital status**				0.45
**Never married**	130(5.85)	94(5.76)	36(4.41)	
**Divorced/Widowed**	367(16.51)	225(14.57)	142(14.76)	
**Married**	1726(77.64)	1103(79.67)	623(80.83)	
**PIR**				**< 0.0001**
**<1.30**	497(22.39)	272(12.18)	225(18.06)	
**1.30-3.49**	793(35.72)	478(27.96)	315(37.95)	
**>=3.50**	930(41.89)	671(59.86)	259(43.99)	
**BMI**				0.05
**Under weight**	12(0.54)	7(0.30)	5(0.42)	
**Normal weight**	517(23.24)	336(22.76)	181(21.21)	
**Over weight**	986(44.31)	657(46.46)	329(39.52)	
**Obese**	710(31.91)	424(30.48)	286(38.85)	
**Smoke status**				**< 0.001**
**Never**	763(34.34)	535(40.76)	228(29.98)	
**Former**	900(40.50)	509(34.28)	391(47.56)	
**Now**	559(25.16)	379(24.96)	180(22.45)	
**Alcohol status**				**< 0.0001**
**Never**	146(6.56)	87(6.20)	59(6.97)	
**Former**	597(26.84)	324(20.06)	273(32.05)	
**Mild**	878(39.48)	563(42.85)	315(42.07)	
**Moderate**	199(8.95)	143(10.15)	56(6.36)	
**Heavy**	404(18.17)	307(20.73)	97(12.54)	
**Creatinine (mg/dl)**	1.03(0.01)	1.01(0.01)	1.06(0.01)	**< 0.001**
**Uric acid (umol/L)**	360.28(2.26)	358.70(2.89)	364.47(3.73)	0.24
**Triglyceride (mmol/L)**	2.65(0.08)	2.72(0.09)	2.48(0.14)	0.17
**Total cholesterol (mmol/L)**	5.38(0.04)	5.46(0.05)	5.17(0.06)	**< 0.001**
**HDL cholesterol (mmol/L)**	1.21(0.01)	1.22(0.01)	1.20(0.01)	0.23
**LDL cholesterol (mmol/L)**	2.98(0.03)	3.03(0.04)	2.87(0.04)	**0.01**
**Diabetes or not**				**< 0.0001**
**Yes**	419(18.83)	171(9.17)	248(28.10)	
**No**	1806(81.17)	1253(90.83)	553(71.90)	
**Hypertension or not**				**< 0.0001**
**Yes**	1129(50.74)	606(38.66)	523(61.31)	
**No**	1096(49.26)	818(61.34)	278(38.69)	
**Benign prostatic hyperplasia**				**< 0.0001**
**Yes**	376(16.9)	158(10.55)	218(25.28)	
**No**	1849(83.1)	1266(89.45)	583(74.72)	
**Prostatitis**				0.44
**Yes**	29(1.3)	15(1.08)	14(1.51)	
**No**	2196(98.7)	1409(98.92)	787(98.49)	
**Prostate cancer**				**< 0.0001**
**Yes**	66(2.97)	11(0.38)	55(5.96)	
**No**	2159(97.03)	1413(99.62)	746(94.04)	

The bold values were used to emphasize the statistical analysis results of P < 0.05, that is, the results were statistically significant.

### 3.3 The relationship between BPH, prostatitis, PCa and ED in NHANES

We conducted weighted multivariate logistic regression models to explore the association between BPH, prostatitis, PCa and ED (see **Table 3**). After adjusting for education level, race, marry status, PIR, smoke status, alcohol status, BMI, creatinine, uric acid, triglyceride, total cholesterol, HDL cholesterol, LDL cholesterol, DM and hypertension (Model 3), we found that BPH was only positively correlated with ED in individuals aged 60-80 (OR=1.93; 95% CI, 1.18-3.18, P=0.02). In contrast, PCa was positively correlated with ED in patients of both age groups. Among individuals aged 40-60, patients with PCa had an 11.90% increased risk of ED compared to those without PCa. Among individuals aged 60-80, patients with PCa had an 7.30% increased risk of ED compared to those without PCa. There was no relationship between prostatitis and ED either in 40-60 age group or 60-80 age group.

**Table 3:** Association between BPH, prostatitis and PCa with ED.

	Model 1		Model 2		Model 3	
	OR(95%CI)	P value	OR(95%CI)	P value	OR(95%CI)	P value
Benign prostatic hyperplasia						
**40-60**	1.46(0.90,2.38)	0.12	1.48(0.86, 2.55)	0.14	1.49(0.77, 2.91)	0.18
**60-80**	1.77(1.24,2.53)	0.002*	1.81(1.22,2.70)	0.01*	1.93(1.18,3.18)	0.02*
Prostatitis						
**40-60**	1.59(0.49,5.11)	0.42	1.99(0.64, 6.15)	0.21	1.76(0.31,10.04)	0.44
**60-80**	2.13(0.40,11.35)	0.36	1.71(0.31,9.59)	0.51	1.42(0.11,18.03)	0.74
Prostatic cancer						
**40-60**	12.32(1.98,76.55)	0.01*	13.24(2.38,73.64)	0.01*	11.90(1.41,100.50)	0.03*
**60-80**	6.49(2.63,15.98)	<0.001*	6.80(2.48,18.63)	0.001*	7.30(2.12,25.08)	0.01*

Model 1: Non-adjusted.

Model 2: Adjusted for education level, race, marry status, PIR, smoke status and alcohol status.

Model 3: Adjusted for education level, race, marry status, PIR, smoke status and alcohol status, BMI, creatinine, uric acid, triglyceride, total cholesterol, HDL cholesterol, LDL cholesterol, diabetes or not and hypertension or not.

*p<0.05

### 3.4 Subgroups analysis in NHANES

**Figure 3:**
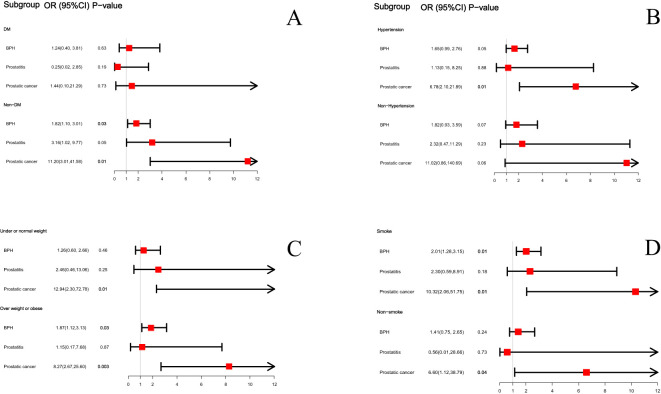
The subgroup analysis between BPH, prostatitis and PCa with ED based on diabetes status **(A)**, hypertension status **(B)**, BMI status **(C)** and smoke status **(D)**.

We conducted the above analysis using subgroup analyses stratified by DM status, hypertension status, BMI levels and smoke status. After adjusting for covariates, BPH was also positively correlated with ED in non-diabetes subgroup (OR=1.82; 95%CI, 1.10-3.01, P=0.03), overweight or obese subgroup (OR=1.87; 95%CI, 1.12-3.13, P=0.03) and in smoke subgroup (OR=2.01; 95%CI, 1.28-3.15, P=0.01). And PCa was positively correlated with ED in non-diabetes subgroup (OR=11.20; 95%CI, 3.01-41.58, P=0.01), hypertension group (OR=6.78; 95%CI, 2.10-21.89, P=0.01), under or normal weight subgroup (OR=12.94; 95%CI, 2.30-72.78, P=0.01), overweight or obese subgroup (OR=8.27; 95%CI, 2.67-25.60, P=0.003), smoke subgroup (OR=10.32; 95%CI, 2.06-51.75, P=0.01) and non-smoke subgroup (OR=6.60; 95%CI, 1.12-38.79, P=0.04). There was no significant difference between BPH, prostatitis, PCa and ED in the DM and Non-hypertension groups and the relationship between prostatitis and ED is not significant in any subgroup (see **Figure 3**).

## 4 Discussion

To our knowledge, this is the premier study which describes the prevalence of ED in groups and estimate the association between BPH, prostatitis, PCa and ED. Moreover, we found BPH and PCa were independent risk factors of ED. The results of subgroup analysis also support the above conclusion. This is particularly important for effective prevention of erectile dysfunction in various populations.

Hormones, particularly testosterone, play a pivotal role in both the development and function of the prostate as well as in regulating sexual function (29). Decreased testosterone levels, which can result as a part of aging or as a consequence of certain treatments for prostate diseases (e.g., androgen deprivation therapy for prostate cancer), are closely associated with both the onset and severity of erectile dysfunction. The testosterone hormonal controls the regulation of erectile physiology by influencing nitric oxide synthesis and penile vascular dynamics, which are critical for erectile response (30). The prostate treatments have impacts on hormonal balance, particularly the effects of surgical and pharmacological treatments for BPH and prostate cancer that may inadvertently lower systemic testosterone levels. Therefore, it is imperative that clinicians monitor hormone levels as part of their management strategy for patients with prostate disease, especially those presenting with ED symptoms.

Prostate diseases, including BPH, and prostate cancer, can significantly impact erectile function through various biological mechanisms. The association between these conditions and ED is primarily mediated by factors such as nerve damage, vascular changes, hormonal imbalances, and inflammatory processes. In the step of multi-factor regression analysis in this study, we find an obvious correlation between BPH and ED among age 60-80 years, which we consider may be related to two clinical factors. The one is that the incidence of BPH reaches the highest after the age of 60, about 79% (31), and the treatment of BPH inevitably affects erectile function. In the medical treatment of BPH, the use of 5-α reductase inhibitors is thought to have a negative effect on the libido in BPH patients, which has been demonstrated in two large placebo control studies (VA study and PROWESS study) (32, 33). Even minimally invasive procedures can inadvertently affect the cavernous nerves running alongside the prostate, which are essential for penile erection (34). In terms of surgical treatment, transurethral resection of prostate (TURP) may damage nerve tracts associated with erectile function in the prostate region, resulting in the incidence of postoperative ED up to 40%. This further leads to the high prevalence of ED in BPH patients (31, 35). The other one is that the sexual need of people aged 60-80, on the basis of the high incidence of BPH, is still higher than that of elderly people aged over 80. The higher emphasis on sexual need has led to more active treatment seeking among men aged 60-80, which has significantly reduced the under-reporting of ED. A survey of 3,015 middle-aged and elderly people which had confirmed this conclusion found that about 73% of people aged 57-64 were sexually active, and the proportion of people aged 65-75 was still as high as 53%, however the figure was only 10% among people aged over 75 (36). Expect the clinical factors, BPH and ED have similar risk factors, suggesting that the pathophysiology of BPH and its underlying mechanisms may be similar to ED. In fact, metabolic status, inflammation, and hormonal environment may play a role in the pathogenesis of BPH and ED (37). Therefore, common treatment strategies for both conditions are currently being explored (38–40). Our research has found a significant relationship between PCa and ED, which is consistent with findings from a cohort study, which revealed that the rate of prescribing ED medication to men diagnosed with prostate cancer increased by 7 times (41). We thought that the impact of prostate cancer on ED was primarily reflected in treatment. A meta-analysis including 890 articles showed that ED was a common complication in prostate cancer patients receiving radiation therapy, and the incidence of ED gradually increased with the increase in radiation did (42). A prospective study indicated that approximately 68% of prostate cancer patients developed ED after undergoing prostatectomy, as revealed by a 24-mon follow-up (43). This may be related to the nerve and tissue damage associated with erectile function after treatment. A retrospective study by R.W.M. Vernooij found that whether bilateral nerves were preserved after surgery was a highly correlated factor in the occurrence of ED after a 24-mon follow-up (44). In addition, hormonal treatment for prostate cancer, such as androgen deprivation therapy (ADT), significantly reduces testosterone levels, which are crucial for erectile function. Lower testosterone levels can lead to decreased libido and ED (45).

Metabolic factors, especially type 2 diabetes, play a central role among the causes of erectile dysfunction (46, 47). Various pathogenic mechanisms may lead to sexual dysfunction in patients with type 2 diabetes, such as alterations in vascular endothelial and smooth muscle function and more (48). Long-standing hyperglycemia associated with diabetes leads to endothelial damage and decreased endothelial nitric oxide synthase (eNOS) activity (49). Nitric oxide (NO) produced by eNOS is essential for the vasodilation necessary for achieving an erection (50). Endothelial dysfunction therefore significantly impairs vascular responses and penile blood flow. At the same time, Diabetes commonly affects peripheral nerves, including those controlling erectile function. Autonomic neuropathy reduces the efficacy of the nerve signals essential for initiating the erectile response (51). Also, certain diabetes medications may negatively affect erectile function (52). Echoing our findings that BPH and PCa were more significantly associated with ED in non-diabetic patients than in diabetic patients, we suggest that this may be due to the independent effect of diabetes on ED, resulting in a less pronounced association between prostate-related diseases and ED in diabetic patients.

In addition to diabetes, we also found that the prevalence of ED was more significant among individuals of hypertension. An observational study of a hypertensive population in the United States revealed that approximately 67-68% of male hypertensive patients experience different degrees of ED (53). Several studies proposed that the continuous and extensive release of vasoconstrictors during hypertension could disturb the equilibrium between vasoconstrictors and vasodilators, which would ultimately lead to adverse effects on vascular and erectile structures (54). Moreover, when it comes to vascular changes, the prostate diseases can lead to vascular changes that affect blood flow to the penis. Conditions like BPH are associated with lower urinary tract symptoms (LUTS), which have been linked to reduced penile blood flow and endothelial dysfunction, thereby impairing the ability to achieve or maintain an erection (11). Compared with previous studies, this study has certain advantages and characteristics. Foremost, we innovatively divided ED into 40-60 and 60-80 age groups to discuss the relationship between BPH, prostatitis, PCa and ED, according to the characteristics of different prevalence rates of ED in different age groups. Secondly, it is the first research based on a large-scale sample size survey design, which enhanced the statistical power of the research and increased the credibility of the research. Thirdly, we have incorporated numerous confounding variables, such as education level, race, marry status, PIR, smoke status and alcohol status, BMI, creatinine, uric acid, triglyceride, cholesterol level, diabetes and hypertension. These variables were not concurrently referenced in initial investigations. Finally, we innovatively subcategorized patients into diabetes, BMI, smoke status and hypertension subgroups to further analyze the significance of the relationship between prostate-related diseases and ED. It is no doubt that our founding holds promising prospects for preventing and managing ED. In the process of diagnosis and treatment of BPH or PCa patients, urological surgeons may strengthen the psychological prevention of ED in patients according to the conclusions of the study.

Prostate diseases, such as BPH and prostate cancer, can significantly impact not only the physical health but also the psychological well-being of affected individuals. The psychological impact of these conditions can, in turn, contribute to the development or exacerbation of ED. The diagnosis and treatment of prostate diseases often lead to significant psychological stress, which can manifest as anxiety or depression. These psychological states are well-known risk factors for erectile dysfunction. The fear of cancer progression, concerns about urinary symptoms, and side effects from treatment can all contribute to a heightened state of anxiety, which can interfere with sexual function (12). Beyond such reasons, the treatments for prostate diseases can lead to changes in body image. Which could alter the dynamics of a patient’s intimate and sexual relationships. The stress of illness can strain relationships, and changes in sexual function can lead to avoidance of intimacy and reduced sexual activity, further exacerbating ED (55).

Our study has some limitations. Firstly, given the reason that data used in the study were all from a cohort of American, more research is needed to investigate whether the conclusions of this study are universally applicable. Thus, more research is needed to confirm whether our findings apply to other ethnic groups. Secondly, in the NHANES database, some information on ED, BPH, prostatitis, PCa and covariates was collected based on self-reported questionnaires, which inherently susceptible to recall bias. Finally, the causal relationship between BPH, PCa and ED would need further research to illustrate due to the cross-sectional design of this study.

## 5 Conclusion

The results of the large cross-sectional study showed a statistically significant association between the BPH, PCa and ED in US adults. Further studies are still needed in the future to validate and replicate our findings and to investigate the specific mechanisms involved.

## Data Availability

The original contributions presented in the study are included in the article/**Supplementary Material**. Further inquiries can be directed to the corresponding authors.
