# Supplementary material for: A comprehensive analysis of erectile dysfunction prevalence and the impact of prostate conditions on ED among US adults: evidence from NHANES 2001-2004
Source: Front Endocrinol (Lausanne). 2025 Jan 13;15:1412369. doi: 10.3389/fendo.2024.1412369 (PMC11769807; doi:10.3389/fendo.2024.1412369)
Supplement: Supplementary file 1 [file DataSheet1.docx]

**Supplementary Table 1 The basic characteristics based on ED or not in 40-60 and 60-80 age group.**

|  | 40-60 | | | 60-80 | | |
| --- | --- | --- | --- | --- | --- | --- |
|  | Non-ED | ED | P value | Non-ED | ED | P value |
| Race |  |  | 0.04* |  |  | 0.48 |
| Mexican American | 196(5.35) | 38(5.04) |  | 77(3.00) | 112(3.27) |  |
| Non-Hispanic Black | 215( 9.14) | 50(11.03) |  | 58(6.48) | 100(7.92) |  |
| Non-Hispanic White | 560(78.47) | 118(72.58) |  | 239(84.13) | 338(83.96) |  |
| Other Hispanic | 32(3.84) | 18(9.33) |  | 8(2.27) | 12(2.46) |  |
| Other Race | 26(3.20) | 5(2.02) |  | 13(4.12) | 10(2.39) |  |
| Education levels |  |  | < 0.001* |  |  | 0.01* |
| Under High School | 93(3.22) | 38(9.86) |  | 73( 8.91) | 143(15.48) |  |
| High School or Equivalent | 369(33.36) | 87(34.95) |  | 143(36.66) | 211(38.08) |  |
| Above High School | 567(63.42) | 104(55.19) |  | 179(54.42) | 218(46.44) |  |
| Marry status |  |  | 0.37 |  |  | 0.08 |
| Never married | 82(6.29) | 23(8.03) |  | 12(3.07) | 13(1.72) |  |
| Divorced/Widowed | 162(15.03) | 34(12.15) |  | 63(12.21) | 108(16.69) |  |
| Married | 783(78.68) | 172(79.81) |  | 320(84.72) | 451(81.59) |  |
| PIR |  |  | 0.003* |  |  | 0.004* |
| <1.30 | 197(12.33) | 70(20.93) |  | 75(11.41) | 155(15.91) |  |
| 1.30-3.49 | 322(26.27) | 71(29.55) |  | 156(36.61) | 244(44.22) |  |
| >=3.50 | 509(61.40) | 88(49.52) |  | 162(51.98) | 171(39.87) |  |
| BMI |  |  | 0.07 |  |  | 0.06 |
| Under weight | 6(0.35) | 1(0.39) |  | 1(0.07) | 4(0.43) |  |
| Normal weight | 244(22.66) | 52(21.54) |  | 92(23.29) | 129(20.97) |  |
| Overweight | 457(45.60) | 85(35.15) |  | 200(50.80) | 244(42.76) |  |
| Obese | 322(31.39) | 91(42.92) |  | 102(25.84) | 195(35.84) |  |
| Smoke status |  |  | 0.20 |  |  | 0.06 |
| Never | 401(42.01) | 70(33.76) |  | 134(34.43) | 158(27.19) |  |
| Former | 315(31.07) | 71(31.85) |  | 194(50.63) | 320(59.20) |  |
| Now | 313(26.92) | 87(34.40) |  | 66(14.94) | 93(13.61) |  |
| Alcohol status |  |  | 0.03* |  |  | 0.06 |
| Never | 57(5.62) | 11(4.31) |  | 30(9.16) | 48(8.96) |  |
| Former | 202(18.41) | 74(30.87) |  | 122(28.48) | 199(32.93) |  |
| Mild | 407(43.17) | 72(37.45) |  | 156(41.27) | 243(45.51) |  |
| Moderate | 108(10.39) | 21( 6.50) |  | 35(8.95) | 35(6.26) |  |
| Heavy | 255(22.42) | 51(20.87) |  | 52(12.15) | 46( 6.34) |  |
| Creatinine (mg/dl) | 1.00(0.01) | 1.00(0.01) | 0.66 | 1.11(0.03) | 1.11(0.02) | 0.88 |
| Uric acid (umol/L) | 357.42(3.11) | 362.36(6.97) | 0.53 | 365.18(3.99) | 366.03(3.61) | 0.89 |
| Triglyceride (mmol/L) | 2.82(0.11) | 2.69(0.27) | 0.66 | 2.19(0.11) | 2.33(0.13) | 0.49 |
| Total cholesterol (mmol/L) | 5.53(0.06) | 5.31(0.10) | 0.07 | 5.10(0.05) | 5.07(0.07) | 0.71 |
| HDL cholesterol (mmol/L) | 1.21(0.01) | 1.18(0.03) | 0.28 | 1.24(0.02) | 1.21(0.02) | 0.26 |
| LDL cholesterol (mmol/L) | 3.06(0.04) | 2.95(0.07) | 0.21 | 2.87(0.05) | 2.81(0.04) | 0.40 |
| Diabetes or not |  |  | <0.0001* |  |  | < 0.001* |
| Yes | 102( 8.20) | 65(27.10) |  | 69(14.13) | 183(28.84) |  |
| No | 927(91.80) | 164(72.90) |  | 326(85.87) | 389(71.16) |  |
| Hypertension or not |  |  | 0.002* |  |  | 0.003* |
| Yes | 369(34.96) | 125(51.49) |  | 237(57.52) | 398(68.60) |  |
| No | 660(65.04) | 104(48.51) |  | 158(42.48) | 174(31.40) |  |
| Benign prostatic hyperplasia |  |  | 0.20 |  |  | 0.004* |
| Yes | 68( 7.75) | 30(10.44) |  | 90(24.81) | 188(36.28) |  |
| No | 961(92.25) | 199(89.56) |  | 305(75.19) | 384(63.72) |  |
| Prostatitis |  |  | 0.72 |  |  | 0.21 |
| Yes | 11(1.18) | 4(1.46) |  | 4(0.58) | 10(1.55) |  |
| No | 1018(98.82) | 225(98.54) |  | 391(99.42) | 562(98.45) |  |
| Prostate cancer |  |  | <0.001* |  |  | < 0.001* |
| Yes | 1(0.09) | 4(1.81) |  | 10(1.84) | 51(9.03) |  |
| No | 1028(99.91) | 225(98.19) |  | 385(98.16) | 521(90.97) |  |
